# Towards a computational model for higher orders of Theory of Mind in social agents

**DOI:** 10.3389/frobt.2024.1468756

**Published:** 2024-10-02

**Authors:** Federico Tavella, Federico Manzi, Samuele Vinanzi, Cinzia Di Dio, Davide Massaro, Angelo Cangelosi, Antonella Marchetti

**Affiliations:** ^1^ Manchester Centre for Robotics and AI, The University of Manchester, Manchester, United Kingdom; ^2^ Department of Computer Science, Faculty of Science and Engineering, The University of Manchester, Manchester, United Kingdom; ^3^ Research Center on Theory of Mind and Social Competence in the Lifespan (CeRiToM), Università Cattolica del Sacro Cuore, Milan, Italy; ^4^ Research Unit on Theory of Mind (UniToM), Department of Psychology, Università Cattolica del Sacro Cuore, Milan, Italy; ^5^ Research Unit on Psychology and Robotics in the Lifespan (PsyRoLife), Department of Psychology, Università Cattolica del Sacro Cuore, Milan, Italy; ^6^ Fondazione Don Carlo Gnocchi Onlus (IRCCS), Milan, Italy; ^7^ Department of Computing, Sheffield Hallam University, Sheffield, United Kingdom

**Keywords:** theory of mind, computational modelling, social agents, perspective taking, artificial intelligence

## Abstract

Effective communication between humans and machines requires artificial tools to adopt a human-like social perspective. The Theory of Mind (ToM) enables understanding and predicting mental states and behaviours, crucial for social interactions from childhood through adulthood. Artificial agents with ToM skills can better coordinate actions, such as in warehouses or healthcare. Incorporating ToM in AI systems can revolutionise our interactions with intelligent machines. This proposal emphasises the current focus on first-order ToM models in the literature and investigates the potential of creating a computational model for higher-order ToM.

## 1 Introduction

We increasingly find ourselves navigating what can be defined as “hybrid societies” (Meyer et al., 2023), where humans interact with artificial agents, and these artificial agents must also interact among themselves, as well as with various humans. All these potential interactions are primarily identified through a social lens (Marchetti et al., 2018) and must adapt to human communication needs (Tavella et al., 2023; Tavella et al., 2024); therefore, artificial tools, to be effective in these societies, should somehow adopt a social interaction perspective that is closer to the human one (Marchetti et al., 2023). Over the last 40 years, one theory that has achieved significant success, both from a cognitive and affective standpoint, is the Theory of Mind (ToM) (Wimmer and Perner, 1983).

ToM is defined as a set of abilities that allow individuals to attribute mental states to themselves and others, and based on these attributions, predict others’ behaviour and plan their own behaviour accordingly (Wellman et al., 1990). Research has extensively demonstrated that ToM is particularly important in managing complex relational situations from early childhood and becomes even more central in adulthood (Wellman et al., 2001; Henry et al., 2013). The development of ToM skills, such as the transition from the first to the second order (Wimmer and Perner, 1983; Perner and Wimmer, 1985) for more details see the ‘Theory of Mind’ section), enables individuals to represent the mental states of multiple agents simultaneously, thus grasping the complexity underlying social relationships.

There are numerous examples that demonstrate how artificial agents and robots could benefit from possessing higher ToM abilities. For instance, multiple artificial agents working in a warehouse (Tubis and Rohman, 2023) could use this skill to understand the current mental states of other agents and use this information to plan their subsequent actions. Another example comes from healthcare: a ToM-equipped robot designed for elderly care (Alameda-Pineda et al., 2024) could consider the perspectives of the elderly and other individuals to provide accurate and appropriate advice (Marchetti et al., 2022). Through these examples, it is evident that ToM not only serves as a critical foundation for personal development and social interaction but also offers a blueprint for enhancing Artificial Intelligence (AI) systems’ capabilities to engage in more human-like interactions. This progression in understanding and applying higher ToM enriches our approach to both developmental psychology and AI, hinting at a future where integrating sophisticated ToM capabilities in artificial agents could profoundly transform our daily interactions with these intelligent machines. For these reasons, scholars strive to develop computational models of ToM to integrate within social robots, aiming to enhance their social understanding and develop a broader spectrum of autonomous behaviours. For instance, Vinanzi et al. (2019) developed a probabilistic model of ToM to assist a humanoid robot in assessing the trustworthiness of a human partner. However, this model is limited to first-order ToM capabilities. If this architecture were expanded to incorporate second-order ToM, the robot would be able to estimate the level of trust another robot places in that human. This advancement could open up scenarios where decision-making is distributed across a network of social robots, each with its own beliefs about the world.

In our proposal, we emphasise the current literature’s focus on first-order ToM models and discuss the potential of a novel methodology to develop a computational model capable of demonstrating higher-order ToM.

## 2 Background

### 2.1 Theory of Mind

From a developmental perspective (Wellman et al., 2001), ToM emerges as a sophisticated ability around the ages of 4–5 years, manifesting in the expression of first-order metarepresentational thinking, i.e., the ability to logically manage different points of view starting from the assumption of recognising one’s mental state. With age, this ability becomes increasingly sophisticated and complex, and around the ages of 6–7 years, we encounter the emergence of what is called second-order competence, precisely defined as a thought of “I think that you think that he thinks”.

The Sally-Anne task is a classic experimental paradigm used to assess first-order metarepresentational thinking, which is crucial for ToM (Wimmer and Perner, 1983). This task highlights the foundation of ToM: the ability to recognise that others can have beliefs, desires, and intentions distinct from one’s own and that may not align with the actual state of the world. Successfully navigating the Sally-Anne task indicates a fundamental level of social cognition, allowing individuals to predict and interpret the behaviour of others based on the understanding of their mental states, a cornerstone of effective social interaction.

Building on the fundamental concept of first-order ToM, as illustrated by the Sally-Anne task, second-order ToM introduces a more complex level of understanding: second-order recursive thinking. This advanced form of cognition involves understanding what one person believes about another person’s beliefs. A classic experiment designed to assess this ability is the Ice Cream Van Task, which serves as a step beyond the simpler Sally-Anne task, requiring a deeper level of metarepresentational thinking (Perner and Wimmer, 1985). Successfully engaging in this task indicates an individual’s capacity to navigate complex social situations involving multiple layers of belief and intention. It reflects an advanced cognitive ability to infer and predict behaviours by considering the interconnected web of beliefs and the perspectives of multiple individuals. Second-order ToM capabilities would enable a social robot to predict a human’s behaviour based on information provided to them by a second human. This recursive thinking would allow the robot to track not only what someone believes but also how that belief was shaped by another person’s perspective, thereby creating new possibilities for human-robot collaboration.

### 2.2 Computational models of Theory of Mind

The current scientific literature presents different computational models of ToM. Here, we briefly describe the distinctive characteristics of these models. It is worth mentioning that all these models share some similarities. For example, all of them are developed and operated within grid-worlds, in which an agent executes actions and a ToM-equipped observer has to make predictions. More specifically, the grid-world contains an agent and several objects that the agent can reach. The task of the observer is to predict both the actions and the goals that will be selected by that agent.

Bayesian Theory of Mind (BToM) (Baker et al., 2011) propose a computational framework aimed at understanding how humans infer the mental states of others, such as beliefs and desires, by observing their actions. BToM conceptualises these inferences through a Partially Observable Markov Decision Process (POMDP), a mathematical framework which incorporates both the observable actions and the unobservable mental states of agents within an environment. It extends the classic Markov Decision Process by incorporating uncertainty in the agent’s perception of the environment, making it more suitable for complex, real-world situations where all relevant information may not be directly accessible. BToM treats the problem of understanding others’ mental states as one of inverse planning: it reconstructs an agent’s mental state, comprising both beliefs about the environment and desires driving their actions, by observing their behaviour in context and applying Bayesian inference. The observer maintains a hypothesis space for the agent’s mental states, evaluating the likelihood of observed behaviour under different combinations of beliefs and desires. BToM was tested through experiments where participants observed agents moving in spatial scenarios, making joint inferences about the agents’ beliefs regarding unseen aspects of the environment and their desires. BToM was later extended to reason on preferences and false beliefs in human-robot interaction settings (Hellou et al., 2023).

Machine Theory of Mind (Rabinowitz et al., 2018) use a meta-learning approach to model ToM. In this scenario, the agents acting into environment models the task as Deep Reinforcement Learning (DRL) problem. By exploring the world and exploiting its experience, the agent learns how to reach its preferred goal. Meanwhile, the observer collect examples of how the agent behaves in the environment and learns in a supervised manner to predict the agent’s actions, goals and beliefs. In particular, the observer is composed by 3 different neural networks: the character network, the mental network and the prediction network. These networks have different tasks, but altogether they create different embedding that when combined model an agent’s mental states. The character network takes care of creating a representation of an agent behaviour based onpast episode trajectories. In turn, the mental net combines such representation with the information about the current episode to infer its mental state. Finally, the prediction net uses the embeddings produced by the character and mental networks to predict the future behaviour of the agent. In particular, the network predicts the probability of taking a certain action, of consuming a certain goal, and successor representations (Dayan, 1993).

Cognitive Theory of Mind (Nguyen and Gonzalez, 2022) build a computational model based on a cognitive theory of decision from experience, namely, Instance-based Learning Theory (IBLT) (Gonzalez et al., 2003). IBLT (Gonzalez et al., 2003) provides not only a decision-making algorithm but also a series of cognitive mechanisms employed in executing computational models. Fundamentally, IBL models derive decisions by extrapolating from prior experiences, called instances, and assessing their similarity to the present decision context. An instance represents a memory unit created from the potential alternatives under consideration. These memory representations consist of three elements: a situation (comprising attributes that contextualise the decision), a decision (the chosen action corresponding to an alternative within the situation), and a utility (the anticipated or actual outcome of the decision within the situation). IBLT leverages cognitive mechanisms from the widely recognised cognitive architecture ACT-R (Ritter et al., 2019) to ascertain how declarative memory elements are accessed, activated, and employed. When presented with an event instance, IBL models utilise three primary mechanisms to reach a decision: activation, probability of retrieval, and blending. Initially, activation value gauges the accessibility of information in memory, considering factors such as similarity and recency. Secondly, activation reflects the likelihood of retrieving an instance from memory. Lastly, blending computes the anticipated utility, leveraging activation and the outcome of the instance. More interestingly, the computational model based on IBLT proposed by Nguyen and Gonzalez (2022) were validated against human participants against the decision making task on the gridworld.

Consequently, we can see how–so far–most researchers focused on models for fist order ToM, but missed the opportunity to address higher orders of ToM.

## 3 Discussion

### 3.1 Proposed methodology

**FIGURE 1:**
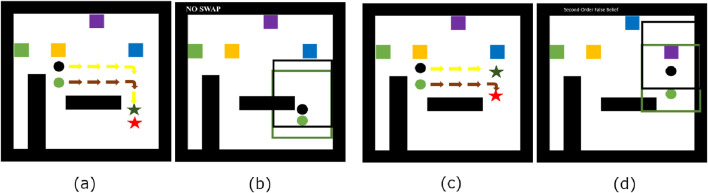
An example of the grid world for the second-order ToM test with two different conditions. In the first scenario – **(A, B)**, – the two agents reach the respective sub, there is no swap of the goal (blue square), neither agents can see the goal and the black agent knows if the green agent can see the goal. In the second scenario – **(C, D)** – the blue and purple goals are swapped and both agents see the swap, with the black agent not knowing the position of the green agent.

We propose to replicate the first-order ToM computational models, scale them to second-order ToM, and validate them through behavioural experiments with humans, such as the user study performed by Nguyen and Gonzalez (2022). We will create a new simulated scenario, similar to the one used in previous experiments, in which two agents, instead of one, must complete a task (see Figure 1). For both agents (black and green), the goal is to reach an intermediate sub-goal, followed by a final goal. The green agent’s sub-goal is strategically positioned to either be within or outside the black agent’s field of vision. The task for the black agent is to position itself between the green agent and its final objective. This setup allows the green agent to observe both the final target position and the black agent’s position within its field of view. Subsequently, with a fixed probability *p*, the target’s position is randomly changed. At this point, we ask our model to predict the black agent’s belief about the green agent’s belief of the target’s position. This approach tests the model’s ability to attribute second-order false beliefs, specifically the black agent’s false belief about the green agent’s belief regarding the target location.

In order to achieve this, we plan on expanding the Machine ToM model. In particular, we select the model developed by Rabinowitz et al. (2018) as it has several interesting properties. First and foremost, it divides past experience (i.e., long-term memory), current trajectories and current states (i.e., working memory) in different inputs for the model. From a psychological standpoint, expanding the Machine ToM model is motivated by the critical role of both long-term and working memory in ToM. Long-term memory allows individuals to retain and process information about past interactions, which is essential for predicting future behaviours and understanding the mental states of others (Perner et al., 2007). The ability to recall past experiences and use this information to infer the beliefs and intentions of others is a cornerstone of effective social cognition. Working memory, on the other hand, is crucial for maintaining and manipulating information in real-time, which is necessary for higher-order ToM (Carlson et al., 2002). This includes understanding what one person thinks about another person’s thoughts, which involves integrating multiple layers of information. This level of complexity in social cognition necessitates advanced working memory capabilities. Secondly, by creating different embeddings for different components of the model, it improves explainability and interpretability regarding the model prediction. Finally, it teaches the model to predict different factors which can influence beliefs in an agent with ToM, such as the next consumed goal, the next action, the successor representation, and beliefs about the goals positions.

**FIGURE 2:**
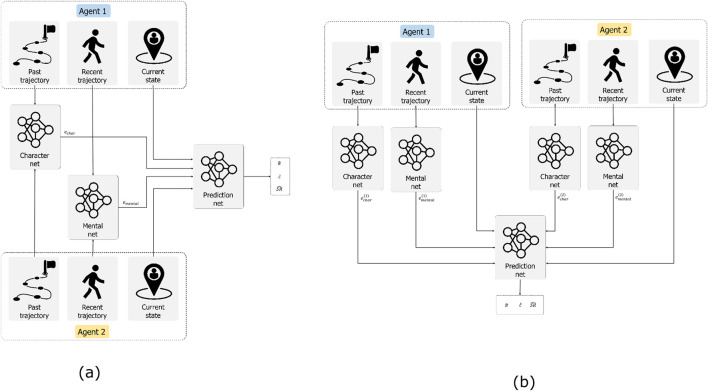
Our two different proposals for the computational model. On the left **(A)**, we propose to adapt the input of the networks to accommodate data from the two different agents. On the right **(B)**, we propose to use separate networks to create different embeddings for different agents, and then use these embeddings for the predictions. Following the notation by Rabinowitz et al. (2018), $\hat{\pi }$ indicates the policy of the agent, $\hat{c}$ the predicted consumed goal, and $\hat{SR}$ the successor representation.

In our opinion, there are two immediate ways of adapting the existing model. On the one hand, we can modify the input so that it takes into account the trajectories of both agents. Moreover, we can modify the output so that it provides predictions for both agents. On the other hand, we could also have two separated models, one for each agent. One model would take care of predicting the behaviour of agent A, while the other would take the output from the previous model, combine it with the information about agent B and provide a prediction about its mental states. In this way, we separate the two “minds” observing the agents. However, at the same time, we duplicate the size of the model, increasing the computational requirements. Figure 2 summarises our two proposed approaches illustrating the two different possibilities, namely, adapting the input or duplicating the model.

### 3.2 Summary and future work

ToM is prospected as a fundamental feature for machines that interact with humans in an hybrid society. In particular, robots need to be able to understand people desires and intentions to interact efficiently and effectively. So far, most of the researchers efforts focused on computational models that simulated ToM up to the first order. However, due to the intricacies of social interactions, this may not be sufficient. We proposed to expand existing computational models beyond the first order by expanding the current architecture proposed by Rabinowitz et al. (2018). Furthermore, we describe how model and test second order ToM with a test inspired by Perner and Wimmer (1985). By doing so, we aim to augment robots with further skills to improve Human-Robot Interaction scenario.

In the future, we aim to develop a computational model that can scale beyond the first order ToM and test its performance again human particupants in the same scenario, as demonstrated in Nguyen and Gonzalez (2022).

## Data Availability

The original contributions presented in the study are included in the article/supplementary material, further inquiries can be directed to the corresponding author.
